# Cereblon deficiency ameliorates carbon tetrachloride-induced acute hepatotoxicity in HepG2 cells by suppressing MAPK-mediated apoptosis

**DOI:** 10.3389/fimmu.2024.1457636

**Published:** 2024-07-30

**Authors:** Seo Young Choi, Parkyong Song, Ji Sun Hwang, You Kyeong Lee, Mi Song Shin, Hong-Joo Son, Yu-Jin Kim, Wanil Kim, Kwang Min Lee

**Affiliations:** ^1^ Department of Life Science and Environmental Biochemistry, Pusan National University, Miryang, Republic of Korea; ^2^ Department of Convergence Medicine, Pusan National University School of Medicine, Yangsan, Republic of Korea; ^3^ New Drug Development Center, Daegu–Gyeongbuk Medical Innovation Foundation, Daegu, Republic of Korea; ^4^ Department of Biochemistry, Department of Convergence Medical Science, and Institute of Medical Science, School of Medicine, Gyeongsang National University, Jinju, Republic of Korea

**Keywords:** inflammation, carbon tetrachloride, pro-inflammatory cytokines, hepatotoxicity, cereblon, MAP kinase, oxidative stress

## Abstract

The liver is vulnerable to various hepatotoxins, including carbon tetrachloride (CCl_4_), which induces oxidative stress and apoptosis by producing reactive oxygen species (ROS) and activating the mitogen-activated protein kinase (MAPK) pathway. Cereblon (CRBN), a multifunctional protein implicated in various cellular processes, functions in the pathogenesis of various diseases; however, its function in liver injury remains unknown. We established a CRBN-knockout (KO) HepG2 cell line and examined its effect on CCl_4_-induced hepatocellular damage. CRBN-KO cells exhibited reduced sensitivity to CCl_4_-induced cytotoxicity, as evidenced by decreased levels of apoptosis markers, such as cleaved caspase-3, and aspartate aminotransferase (AST) and alanine aminotransferase (ALT) activities. CRBN deficiency enhanced antioxidant defense, with increased superoxide dismutase activity and glutathione ratios (GSH/GSSG), as well as reduced pro-inflammatory cytokine expression. Mechanistically, the protective effects of CRBN deficiency appeared to involve the attenuation of the MAPK-mediated pathways, particularly through decreased phosphorylation of JNK and ERK. Overall, these results suggest the crucial role of CRBN in mediating the hepatocellular response to oxidative stress and inflammation triggered by CCl_4_ exposure, offering potential clinical implications for liver injury in a wide range of liver diseases.

## 1 Introduction

The human liver plays a crucial role in the detoxification and metabolic conversion of chemicals, drugs, and harmful substances (1). Detoxification generates reactive oxygen species (ROS) that contribute to oxidative damage, cellular disturbance, and hepatotoxicity. Excessive ROS levels pose a significant risk of cellular toxicity, resulting in oxidative stress (2).

Oxidative stress exhibits several detrimental effects on cells. Oxidized proteins become vulnerable to proteasomal degradation, and reactive oxygen species (ROS) disrupt mitochondrial integrity, altering permeability and releasing pro-apoptotic factors, such as cytochrome C (3). Increased mitochondrial permeability activates caspase-3, leading to cell death (4). Oxidative stress, resulting from an imbalance between ROS production and antioxidant defense mechanisms, is implicated in various diseases, including cancer, arteriosclerosis, diabetes, neurodegeneration, and acute and chronic liver conditions (5–7).

Carbon tetrachloride (CCl_4_) is a hepatotoxin frequently used to induce liver damage in experimental models. In the liver, CCl_4_ induces free radical generation via metabolic processes. CCl_4_ undergoes metabolic activation through a cytochrome P-450-dependent process, further leading to the production of free radicals that initiate inflammation (8). CCl_4_ also causes the peroxidation of cellular and organelle membrane lipids, inhibits protein synthesis, and disrupts cell morphology, ultimately culminating in apoptosis and necrosis of hepatocytes (9, 10). Thus, understanding the mechanisms of cellular antioxidant defenses is crucial for mitigating the liver damage caused by oxidative stress.

Cereblon (CRBN) is a multifunctional protein that plays diverse roles in cellular physiology and disease pathogenesis. Initially identified as a gene associated with mild forms of autosomal recessive non-syndromic mental retardation (ARNSMR) (11), CRBN has been implicated in various cellular processes (12–14). CRBN is a target for thalidomide-induced birth defects and a substrate receptor within the Cullin-RING E3 ubiquitin ligase (CRL) complex (15). Through direct interaction with the alpha subunit of AMP-activated protein kinase (AMPK, a pivotal metabolic sensor), CRBN inhibits the enzymatic activity of AMPK, suggesting its potential role in metabolic disorders (16, 17). The dysregulation of CRBN function has been implicated in various diseases, including cancer, neurodegenerative disorders, and developmental abnormalities (18–20). However, the physiological role(s) of CRBN in liver injury and its underlying molecular mechanisms have not been investigated yet.

In this study, we investigated the potential hepatoprotective role of CRBN in hepatocellular injury. We specifically explored the effect of CRBN knockout on CCl_4_-induced liver damage in HepG2 cells and the underlying molecular pathways through which CRBN mitigates hepatocellular injury. Our findings provide insights into novel therapeutic approaches for liver diseases in multiple pathological contexts.

## 2 Materials and methods

### 2.1 Cell culture and CCl_4_ treatment

HepG2 human hepatoblastoma cell line was purchased from the Korean Cell Line Bank (Seoul, South Korea) and cultured in Dulbecco’s modified Eagle’s medium (DMEM; GIBCO, Waltham, USA) supplemented with 10% heat-inactivated fetal bovine serum (FBS; Hyclone), 100 units/mL penicillin, and 100 µg/mL streptomycin sulfate. α-Mouse liver 12 (AML12; immortalized mouse hepatocytes) cell line was a kind gift of Prof. Hueng-Sik Choi (School of Biological Sciences and Technology, Chonnam National University, Republic of Korea) and cultured in DMEM/F-12 medium (Gibco, Eggenstein, Germany) supplemented with 10% heat-inactivated FBS, 0.1% insulin-transferrin-selenium (ITS; Sigma, Burligton, USA), 40 ng/ml dexamethasone (Sigma, USA), 100 units/mL penicillin, and 100 µg/mL streptomycin sulfate. The cells were maintained at 37°C in a humidified atmosphere containing 5% CO2. For the following experiments, the cells were seeded in a 12-well plate and allowed to reach 90% confluency. Subsequently, the cells were treated with 1 mL of the indicated concentrations of CCl_4_ dissolved in DMEM containing 0.25% DMSO (21). The control treatment included only DMEM containing 0.25% DMSO. After 1 h of CCl_4_ treatment, the cells and culture medium were collected separately. The cells were lysed with RIPA buffer for western blot analysis, homogenized according to the manufacturer’s protocol for measuring SOD activity and GSH/GSSG ratio, and total RNA was extracted using TRIzol reagent. The culture medium was used to measure the AST and ALT activities.

### 2.2 Generation of CRBN knockout cell line using CRISPR-Cas9-mediated gene editing

The CRISPR/Cas9 Gene Knockout Kit specific for the human CRBN gene (sc-418922) or control (sc-412142) was purchased from Santa Cruz Biotechnology (Santa Cruz, CA, USA). Plasmids containing the CRISPR/Cas9 system were transfected into HepG2 cells using the Lipofectamine transfection reagent (Invitrogen, Carlsbad, CA, USA), following the manufacturer’s instructions. Two days after transfection, the cells were treated with 2 μg/mL puromycin (Sigma, St. Louis, MO, USA) for 3 days to select for transfected cells. Subsequently, serial dilutions were performed in 96-well plates to obtain one cell per well for clonal selection. The expression of CRBN was analyzed using western blotting.

### 2.3 AST and ALT activity

AST and ALT activities in the supernatant after exposure of cells to 0.1% CCl_4_ were determined using the colorimetric assay kit (AST; #K753-100, ALT; #K752- 100, Biovision Co., CA, USA), according to manufacturer’s instructions.

### 2.4 Antioxidant parameters

SOD activity and the GSH/GSSG ratio were determined using commercially available assay kits (SOD; #S311, GSH/GSSG; #G257, Dojindo Molecular Technologies, Kumamoto, Japan), according to the manufacturer’s instructions.

### 2.5 Western blotting

Proteins were separated using sodium dodecyl sulfate-polyacrylamide gel electrophoresis and transferred onto polyvinylidene fluoride membranes. After blocking with 3% BSA in TBS-T (137 mM NaCl, 20 mM Tris-Cl, pH 7.6, 0.1% Tween 20), the membranes were incubated with various primary antibodies, including anti-cleaved caspase-3 (cell signaling 9664), anti-CRBN (cell Signaling 71810), anti (MAPK; Erk1/2) (R&D systems MAB1576), anti-phospho-p44/42 MAPK (Erk1/2) (cell signaling 4377), anti-p38 MAPK (cell signaling 8690), anti-phospho-p38 MAPK (cell signaling 4511), anti-JNK (cell signaling 9252), anti-phospho-JNK (cell signaling 4668), and anti-tubulin (Sigma-Aldrich T6199). The blots were then incubated with a secondary antibody [anti-rabbit horseradish peroxidase-conjugated or anti-mouse horseradish peroxidase conjugate; Santa Cruz Biotechnology)], and the protein bands were visualized using an enhanced chemiluminescence detection system.

### 2.6 Cell viability assay

Cell viability was measured using the water-soluble tetrazolium salt 1 assay (WST-1; EZ-CyTox, Dogen, Korea), according to the manufacturer’s instructions. The optical density of the control cells was considered 100% viable.

### 2.7 Quantitative realtime PCR

*IL-1β*, *IL-6*, *TNF-α*, and *COX-2* gene expression was quantified using quantitative real-time polymerase chain reaction (qRT-PCR). Briefly, the total RNA of HepG2 cells was extracted using TRIzol reagent (Life Technologies, Carlsbad, CA, USA). Total RNA (2 µg) was used as a template for synthesizing the first-strand of cDNA using the reverse transcriptase reaction mixture (Takara, Shiga, Japan). For qRT-PCR, the PCR mix (FastStart Universal SYBR Green Master; Roche, Switzerland) used in the reaction system and thermocycler conditions were set according to the manufacturer’s instructions. Relative expression data were normalized to GAPDH and are presented as a ratio relative to the control. The primer sequences used for the PCR analyses are listed in **Table 1**.

**Table 1:** Oligonucleotide sequences used in RT-PCR analysis.

Gene	Direction	Sequence (5′to 3′)
**IL-1β**	Forward Reverse	CCACAGACCTTCCAGGAGAATG GTGCAGTTCAGTGATCGTACAGG
**IL-6**	Forward Reverse	ACTCACCTCTTCAGAACGAATTG CCATCTTTGGAAGGTTCAGGTTG
**TNF-α**	Forward Reverse	CTCTTCTGCCTGCTGCACTTTG ATGGGCTACAGGCTTGTCACTC
**COX-2**	Forward Reverse	TGCATTCTTTGCCCAGCACT AAAGGCGCAGTTTACGCTGT
**GAPDH**	Forward Reverse	GACTCATGACCACAGTCCATGC AGAGGCAGGGATGATGTTCTG

### 2.8 Statistical analysis

Significant differences between groups were determined using two-tailed unpaired Student’s t-tests, and multiple comparisons were performed using one-way or two-way repeated-measures ANOVA. Data are expressed as the mean ± standard error of the mean (SEM) of at least three independent experiments. Statistical significance was set at P < 0.05, as indicated in the figure legends.

## 3 Results

### 3.1 Dose-dependent cytotoxicity of carbon tetrachloride in HepG2 cells

**Figure 1:**
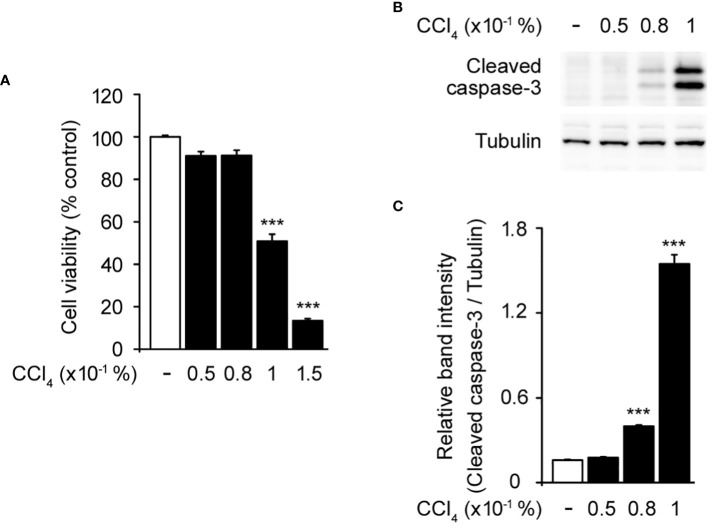
Toxic effects of carbon tetrachloride (CCl_4_) on HepG2 cells. **(A)** Cytotoxicity of CCl_4_ on HepG2 cells. **(B)** Western blot of the cleaved caspase-3 in HepG2 cells. **(C)** Relative band intensity of cleaved caspase-3 quantified using densitometric analysis and then normalized to that of tubulin. HepG2 cells were incubated with different concentrations of CCl_4_ (0.05, 0.08, 0.1, and 0.15%) for 1 h. Values in the bar graphs are the mean ± SEM of at least three independent experiments. ***P < 0.005, significantly different from the control group.

To establish a baseline for understanding the effect of CCl_4_ on hepatocytes, we initially assessed the cytotoxicity of various concentrations of CCl_4_ in HepG2 cells, a human liver cancer cell line that is used to model hepatocyte function *in vitro* (22, 23). This was crucial for determining the effective dose that induced observable cellular damage without immediate lethality, thereby facilitating subsequent investigations into the protective role of CRBN. Treatment with 0.05–0.08% CCl_4_ did not significantly affect cell viability compared to the untreated controls. However, 0.1% CCl_4_ exhibited a significant reduction in cell viability, establishing this as the optimal concentration for inducing a measurable and consistent toxic effect (**Figure 1A**). We confirmed these effects by measuring the activity of caspase-3, an indicator of apoptosis, using western blotting (24). Increased cleaved caspase-3 levels were evident at 0.1% CCl_4_ (**Figures 1B, C**).

### 3.2 CRBN deficiency prevented CCl_4_-stimulated hepatotoxicity *in vitro*

**Figure 2:**
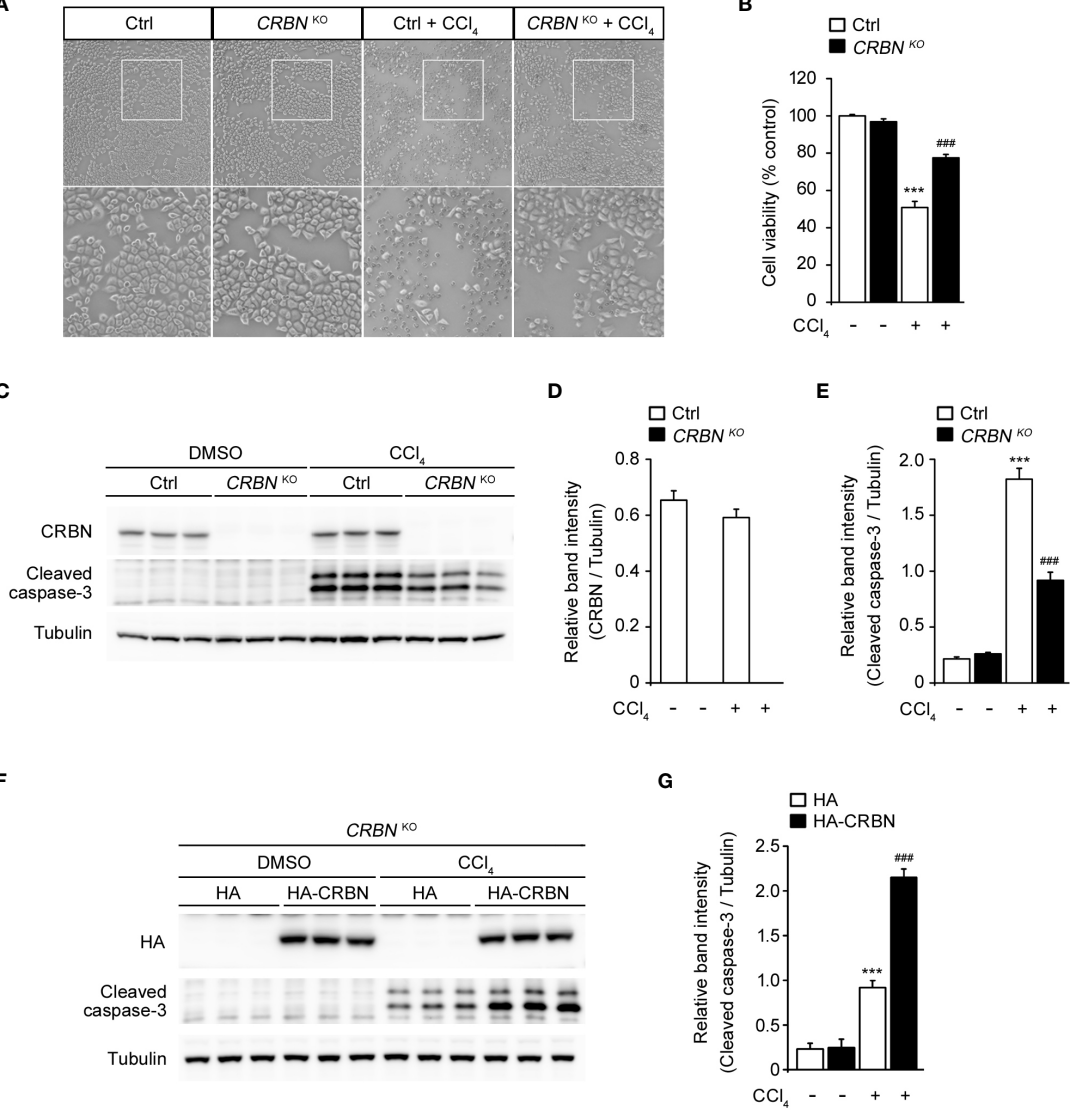
Effect of CRBN knockout (KO) on CCl_4_-induced damage in HepG2 cells. **(A)** Representative microscopic images of HepG2 cells (Ctrl and CRBN KO) treated with DMSO or CCl_4_, respectively. The regions outlined in white in the upper images are shown in the bottom panels. Original magnification, ×100. **(B)** Relative cell viability presented using the WST-1 of the cells from **(A)**. **(C)** Representative western blotting analysis of CRBN, cleaved caspase-3, and tubulin in HepG2 cells (Ctrl and CRBN KO) treated with either DMSO or CCl_4_. Tubulin was used as a loading control. **(D)** Relative band intensity of CRBN quantified using densitometric analysis and then normalized to that of tubulin. **(E)** Relative band intensity of cleaved caspase-3 quantified using densitometric analysis and then normalized to that of tubulin. **(F)** Representative Western blotting analysis of HA, cleaved caspase-3, and tubulin in CRBN KO treated with either DMSO or CCl_4_. Tubulin was used as a loading control. **(G)** Relative band intensity of cleaved caspase-3 was quantified using densitometric analysis and then normalized to that of tubulin. Ctrl-and CRBN KO HepG2 cells were treated with either DMSO or CCl_4_ (0.1%) for 1 h. The values in the bar graphs represent the mean ± SEM of at least three independent experiments. *Statistical differences (*P < 0.05, **P < 0.01, ***P < 0.005) compared to the control HepG2 cells not treated with CCl_4_. ^#^Statistical differences (^#^P < 0.05, ^##^P < 0.01, ^###^P < 0.005) compared to the control HepG2 cells treated with CCl_4_.

To investigate the functional involvement of CRBN in liver injury, we used CRISPR-Cas9 technology to develop CRBN knockout (CRBN KO) and control (Ctrl) HepG2 cells. The cells were treated with CCl_4_ and its morphological changes were evaluated with inverted microscopy and quantitative analysis of cell viability using the WST-1 assay. CCl_4_ treatment significantly inhibited cell confluence and caused cell death in control cells, which is in agreement with previous studies (1, 25, 26). However, CCl_4_-induced morphological alterations were prevented in the CRBN KO HepG2 cells (**Figure 2A**). A comparison of cell viability between Ctrl and CRBN KO cells in the presence of CCl_4_ revealed that CRBN KO cells exhibited improved viability against CCl_4_-induced cytotoxicity (**Figure 2B**).

Thus, we next performed western blotting for the protein expression of cleaved caspase-3 to confirm the resistance to cell death conferred by CRBN KO (**Figure 2C**). CCl_4_-treated CRBN KO cells showed a significantly decreased cleaved caspase-3 level compared with CCl_4_-treated ctrl cells (**Figures 2C, E**). Notably, endogenous CRBN expression was not affected by CCl_4_ treatment (**Figures 2C, D**). These results suggested that CRBN is an essential component of apoptotic signaling in CCl_4_-induced hepatocellular injury *in vitro*.

To further validate the functional role of CRBN in CCl_4_-stimulated hepatotoxicity, we attempted to rescue the phenotype of the CRBN deficiency by exogenously expressing HA-tagged CRBN in CRBN KO HepG2 cells followed by CCl_4_ treatment (**Figure 2F**). Western blotting revealed that overexpression of CRBN markedly increased the levels of cleaved caspase-3 in CRBN KO cells compared to mock-transfected CRBN KO cells treated with CCl_4_ (**Figure 2G**), further confirming the critical role of CRBN in mediating CCl_4_-induced hepatocellular injury and indicating that the observed protective effects are attributable to CRBN deficiency.

### 3.3 CRBN deletion ameliorated CCl_4_-induced oxidative stress in HepG2 cell lines

**Figure 3:**
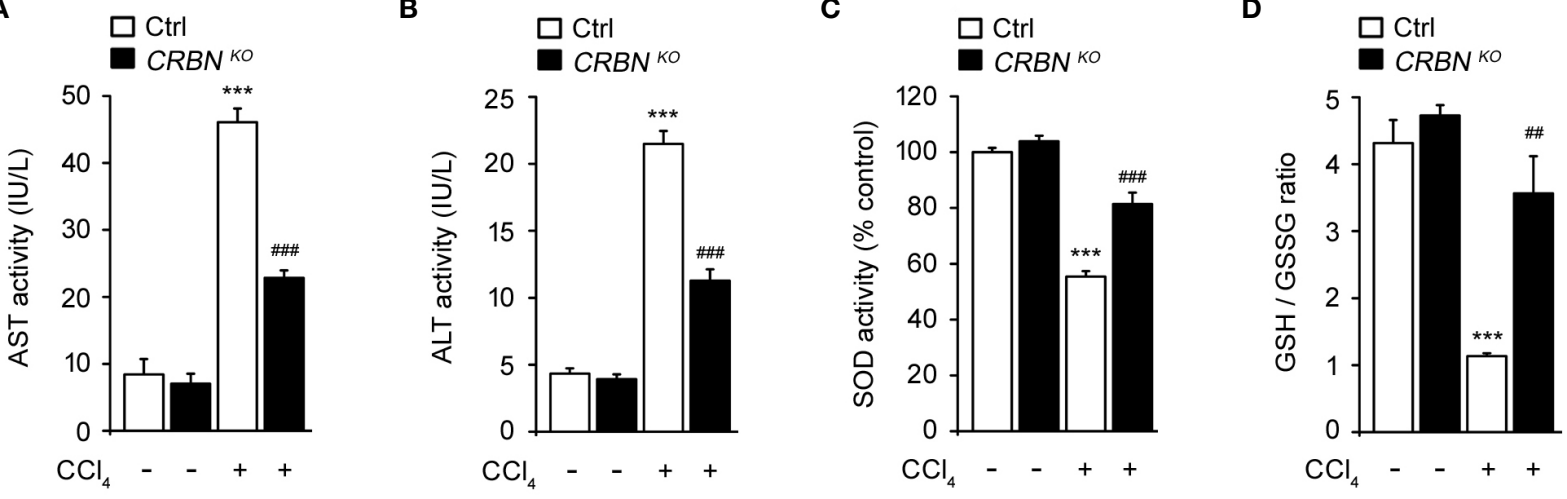
Effect of CRBN loss on the biochemical parameters and antioxidant enzyme activity of CCl_4_-treated HepG2 cells. **(A)** AST activity of HepG2 cells. **(B)** ALT activity of HepG2 cells. **(C)** SOD activity. **(D)** GSH/GSSG ratio. Ctrl-and CRBN KO HepG2 cells were treated with either DMSO or CCl_4_ (0.1%) for 1 h. The values in the bar graphs represent the mean ± SEM of at least three independent experiments. *Statistical differences (*P < 0.05, **P < 0.01, ***P < 0.005) compared to the control HepG2 cells not treated with CCl_4_. ^#^Statistical differences (^#^P < 0.05, ^##^P < 0.01, ^###^P < 0.005) compared to the control HepG2 cells treated with CCl_4_.

To clarify the protective effect of CRBN KO against hepatocellular injury, we examined aspartate aminotransferase (AST) and alanine aminotransferase (ALT) activity in HepG2 cells after exposure to CCl_4_. AST and ALT are enzymes primarily found in hepatocytes and are released into the bloodstream upon liver injury. Therefore, measuring AST and ALT activity serves as a sensitive and reliable method for assessing hepatocellular injury (8). CCl_4_-treated ctrl cells exhibited markedly elevated AST and ALT activities compared with CCl_4_ untreated ctrl cells (**Figures 3A, B**). This observation is consistent with previous studies that demonstrated the hepatotoxic effects of CCl_4_ and the release of AST and ALT into the culture media as markers of hepatocellular injury (8, 27). In contrast, CRBN KO HepG2 cells treated with CCl_4_ exhibited a notable reduction in AST and ALT activities compared with control HepG2 cells treated with CCl_4_.

Oxidative stress is essential in the progression of liver injury. Intracellular antioxidants, such as superoxide dismutase (SOD) and glutathione (GSH), play crucial roles in protecting the liver from oxidative stress-induced damage by neutralizing harmful reactive oxygen species (ROS) (28). Thus, SOD activity and the GSH/GSSG ratio were also examined. CCl_4_-treated ctrl cell showed a notable decrease in SOD activity and the GSH/GSSG ratio compared with CCl_4_ untreated ctrl cells (**Figures 3C, D**). These reductions in SOD activity and the GSH/GSSG ratio indicate an imbalance between ROS production and antioxidant defense mechanisms, resulting in oxidative stress and cellular damage caused by CCl_4_ treatment. In contrast, CRBN deficiency group exposed to CCl_4_ exhibited the preservation of endogenous SOD activity and the GSH/GSSG ratio compared with the control group exposed to CCl_4_.

These findings further support the protective role of CRBN KO against hepatocellular damage, implying a potential mechanism by which CRBN modulates oxidative stress and protects against liver injury.

### 3.4 CRBN deficiency resulted in the defective expression of the genes involved in CCl_4_-induced inflammation

**Figure 4:**
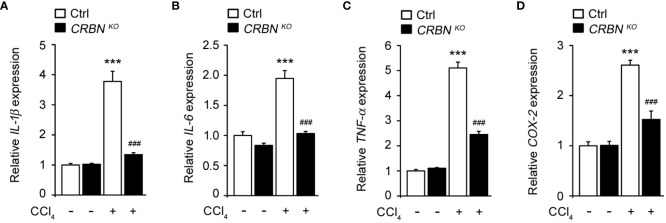
Effect of CRBN KO on pro-inflammatory cytokine mRNA expression in the HepG2 cells treated with CCl_4_. Total RNA was isolated from HepG2 cells (Ctrl and CRBN KO) treated with DMSO or CCl_4_, and subjected to quantitative real-time PCR analysis to determine the expression of **(A)** *IL-1β*, **(B)** *IL-6*, **(C)** *TNF-α, and* **(D)** *COX-2*. Expression levels were normalized to GAPDH mRNA levels. Fold changes in mRNA levels relative to that of the control HepG2 cells not treated with CCl_4_, which were set arbitrarily at 1.0, are shown. The values in the bar graphs represent the mean ± SEM of at least three independent experiments. *Statistical differences (*P < 0.05, **P < 0.01, ***P < 0.005) compared to the control HepG2 cells not treated with CCl_4_. ^#^Statistical differences (^#^P < 0.05, ^##^P < 0.01, ^###^P < 0.005) compared to the control HepG2 cells treated with CCl_4_.

Inflammation plays a critical role in hepatic injury progression. To evaluate the effect of CRBN deficiency on CCl_4_-induced inflammation, we assessed the mRNA levels of proinflammatory genes, including *IL-1b*, *IL-6*, *TNF-a*, and *COX-2*, in cells following CCl_4_ exposure. CCl_4_-treated ctrl cells showed a significant upregulation in *IL-1b*, *IL-6*, *TNF-a*, and *COX-2* expression compared with CCl_4_ untreated ctrl cells (**Figure 4**). This demonstrated that CCl_4_-induced pro-inflammation, indicating an inflammatory response triggered by hepatocellular injury. Conversely, CRBN KO HepG2 cells treated with CCl_4_ exhibited a notable decrease in *IL-1b*, *IL-6*, *TNF-a*, and *COX-2* mRNA levels compared with control HepG2 cells treated with CCl_4_. These results demonstrated that CRBN deletion attenuated the upregulation of pro-inflammatory genes induced by CCl_4_ exposure in HepG2 cells, suggesting the importance of CRBN in modulating inflammatory responses in the liver.

### 3.5 CRBN KO attenuated CCl_4_-induced mitogen-activated protein kinase (MAPK) pathway

Mitogen-activated protein kinases (MAPKs) are pivotal regulators of cellular processes and play critical roles in proliferation, apoptosis, and immune defense (29). MAPKs form three distinct pathways in mammalian cells, the ERK, JNK kinase, and p38 pathways. Each pathway responds to specific extracellular signals, and regulates various cellular processes (30). CCl_4_-induced oxidative stress initiates apoptosis via the activation of the MAPK family (31–33). Therefore, we investigated the function of the MAPK pathway in the molecular mechanisms underlying the protective effects of CRBN knockout against CCl_4_-induced cell damage.

**Figure 5:**
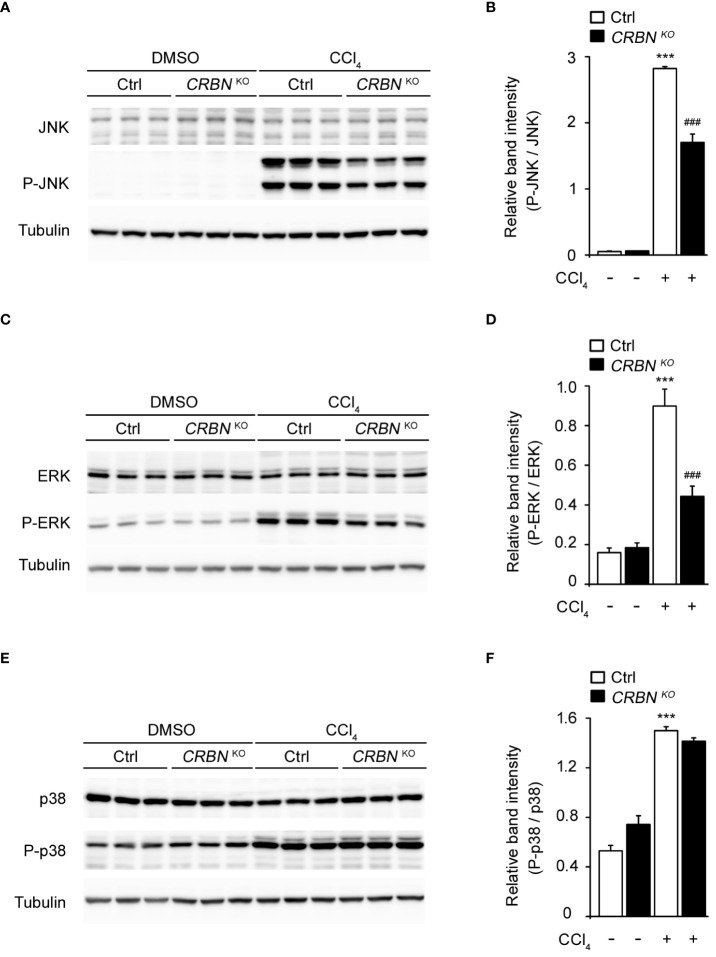
Effect of CRBN deletion on ERK 1/2, JNK 1/2, and p38 expression in the HepG2 cells treated with CCl_4_. **(A)** Representative western blotting analysis of JNK and P-JNK. Tubulin was used as the loading control. **(B)** Relative band intensity of P-JNK quantified using densitometric analysis and then normalized to that of JNK. **(C)** Representative western blotting analysis of ERK and P-ERK. **(D)** Relative band intensity of P-ERK to ERK. **(E)** Representative western blotting analysis of p38 and P-p38. **(F)** Relative band intensity of P-p38 to p38. Ctrl-and CRBN KO HepG2 cells were treated with DMSO or CCl_4_. The values in the bar graphs represent the mean ± SEM of at least three independent experiments. *Statistical differences (*P < 0.05, **P < 0.01, ***P < 0.005) compared to the control HepG2 cells not treated with CCl_4_. ^#^Statistical differences (^#^P < 0.05, ^##^P < 0.01, ^###^P < 0.005) compared to the control HepG2 cells treated with CCl_4_.

CCl_4_ treatment of control cells significantly increased the levels of phospho-JNK, phospho-ERK, and phospho-p38 MAPK proteins compared with CCl_4_-untreated control cells (**Figure 5**), consistent with previous reports (34, 35). However, the absence of CRBN decreased the CCl_4_-induced phosphorylation of JNK and ERK, whereas the phosphorylation of p38 MAPK remained unaffected. (**Figure 5**). Notably, the total protein levels of the three MAPKs did not change, regardless of CCl_4_ treatment or CRBN knockout. These observations indicate that CRBN knockout attenuates the activation of JNK and ERK in response to CCl_4_-induced oxidative stress, but not that of p38 MAPK, suggesting a molecular mechanism underlying the protective effects of CRBN knockout against CCl_4_-induced cell damage and the potential involvement of the MAPK pathway in mediating these effects.

### 3.6 CRBN knockdown mitigated CCl_4_-induced hepatic toxicity in AML12 cells

To further validate the protective role of CRBN deficiency against CCl_4_-induced hepatocellular injury, we extended our study to another cell line using TD-165, a well characterized CRBN degrader (18, 36) in AML-12 cells, which are derived from mouse hepatocytes.

**Figure 6:**
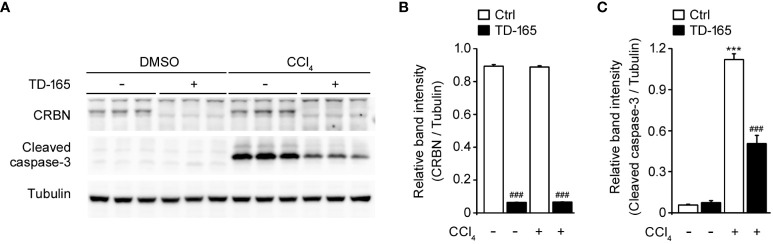
Effects of CRBN knockdown (KD) against CCl_4_-induced damage in AML12 cells. AML12 cells were treated with or without TD-165 (1μM) for 24 h and subsequently exposed to CCl_4_ (0.1%) for an additional 1 h. Cell lysates were analyzed by Western blot. **(A)** Representative Western blotting analysis of CRBN, cleaved caspase-3, and tubulin. Tubulin was used as a loading control. **(B)** Relative band intensity of CRBN was quantified using densitometric analysis and then normalized to that of tubulin. **(C)** Relative band intensity of cleaved caspase-3 was quantified using densitometric analysis and then normalized to that of tubulin. *Statistical differences (*P < 0.05, **P < 0.01, ***P < 0.005) compared to the DMSO control group without TD-165. ^#^Statistical differences (^#^P < 0.05, ^##^P < 0.01, ^###^P < 0.005) compared to the corresponding treatment group without TD-165.

We first confirmed the effectiveness of the CRBN degrader by assessing CRBN expression levels. Treatment with TD-165 significantly reduced CRBN expression in AML12 cells, demonstrating successful knockdown (**Figures 6A, B**).

To examine the impact of CRBN knockdown against CCl_4_-induced hepatic toxicity, we measured the levels of cleaved caspase-3. Western blot analysis revealed that CCl_4_-treated CRBN knockdown AML12 cells showed a significantly decreased cleaved caspase-3 level compared with CCl_4_-treated ctrl AML12 cells (**Figure 6C**), consistent with our findings in HepG2 cells. These results suggest that the protective role of CRBN deficiency against CCl_4_-induced hepatic toxicity is not limited to HepG2 cells and can be replicated in AML12 cells, further supporting the protective role of CRBN deficiency against hepatocellular injury.

## 4 Discussion

The liver is a prime target for reactive oxygen species (ROS), and it possesses an intricate antioxidant system that preserves redox balance within its tissues. Elevated ROS levels can disrupt this balance, leading to oxidative stress, which is heavily implicated in liver diseases and various chronic and degenerative conditions (9, 37). In a CCl_4_-induced liver injury model, the triggered oxidative stress leads to lipid peroxidation that damages the membranes of hepatocytes. This damage results in a significant release of pro-inflammatory chemokines and cytokines, ultimately exacerbating liver damage (38). Given its ability to mimic chronic liver disease, CCl_4_ has been commonly used as a hepatotoxin in numerous experimental models (39–41).

CRBN performs multifunctional roles in cellular physiology and in a wide array of human diseases (42–44). However, the hepatoprotective role of CRBN against CCl_4_-induced hepatic injury remains unknown. Our study demonstrated that CRBN KO may confer protection against CCl_4_-induced acute liver injury, potentially through mechanisms involving the inhibition of inflammatory responses and attenuation of oxidative stress.

CRBN KO cells exhibited improved viability and reduced apoptosis in response to CCl_4_ treatment compared with control cells. The observed decrease in caspase-3 activation, a hallmark of apoptosis, along with lower AST and ALT activities, suggests a substantial reduction in hepatocellular damage. These findings not only underline the potential hepatoprotective attributes of CRBN modulation, but also advance our understanding of the molecular interactions involved in liver toxicity.

The reduced pro-inflammatory cytokine expression in CRBN-KO cells following CCl_4_ treatment also highlights the important anti-inflammatory effects of CRBN deletion. Given the role of inflammation in the exacerbation of liver damage, the ability to suppress inflammatory mediators can be highly beneficial in clinical settings where inflammation significantly contributes to disease progression (45). Additionally, we explored antioxidant defense mechanisms by examining SOD activity and the GSH/GSSG ratios. The preservation of these antioxidants in the CRBN-KO cells further supports the role of CRBN in oxidative stress, which is a key player in the pathogenesis of liver injury. By maintaining a balance between ROS production and antioxidant defense, CRBN-KO cells are better equipped to reduce oxidative stress and the likelihood of cellular damage and death. Our study also revealed that the protective mechanism likely involved the modulation of MAPK signaling pathways. Specifically, the reduced phosphorylation of JNK and ERK in CRBN KO cells treated with CCl_4_ compared with that in ctrl cells treated with CCl_4_ indicates a targeted disruption of these pathways, which mediate cellular responses to stress and damage. Interestingly, p38 MAPK phosphorylation remained unchanged, suggesting the selective involvement of certain MAPK pathways in the protective effects conferred by CRBN deletion. This selectivity may be pivotal for therapeutic strategies aimed at minimizing hepatocellular injury without interfering with other cellular functions regulated by p38 MAPK.

The rescue experiment, where re-expression of CRBN in CRBN KO HepG2 cells reversed the protective effects, further confirms the critical role of CRBN in mediating CCl_4_-induced hepatocellular injury. Additionally, our observations in AML12 cells suggest that the protective role of CRBN deficiency extends across different hepatocyte models, indicating the broader applicability of CRBN’s protective effects against CCl_4_-induced hepatotoxicity.

Our study conclusively demonstrates the hepatoprotective capabilities of CRBN in CCl_4_-induced hepatocellular injury. Specifically, we found that CRBN plays a critical role in mitigating damage, at least in part, through mechanisms that reduce oxidative stress and inflammation, and through the inhibition of MAPK pathway-induced apoptosis. Although further animal studies or clinical trials are needed to validate our findings and enhance the translational potential of our results, the present study provides deeper insights into the molecular mechanisms of liver disease and opens new avenues for targeted therapies to prevent or mitigate liver injury in a range of pathological contexts.

In conclusion, the present study not only highlights the protective role of CRBN deletion against CCl_4_-induced hepatocellular injury, but also provides a foundation for further investigations into gene-targeted therapies for liver injury across a spectrum of liver diseases.

## Data availability statement

The original contributions presented in the study are included in the article/supplementary material. Further inquiries can be directed to the corresponding authors.

## Ethics statement

Ethical approval was not required for the studies on humans in accordance with the local legislation and institutional requirements because only commercially available established cell lines were used. Ethical approval was not required for the studies on animals in accordance with the local legislation and institutional requirements because only commercially available established cell lines were used.

## Author contributions

SC: Writing – original draft, Validation, Investigation, Formal analysis, Data curation. PS: Writing – review & editing, Validation, Investigation, Formal analysis, Data curation. JH: Writing – review & editing, Validation, Formal analysis, Data curation. YL: Writing – review & editing, Resources, Formal analysis. MS: Writing – review & editing, Resources, Data curation. H-JS: Writing – review & editing, Resources. Y-JK: Writing – review & editing, Resources. WK: Writing – review & editing, Writing – original draft, Supervision, Data curation, Conceptualization. KL: Writing – review & editing, Writing – original draft, Validation, Supervision, Investigation, Data curation, Conceptualization.
